# Reduced Serotonin Reuptake Transporter (SERT) Function Causes Insulin Resistance and Hepatic Steatosis Independent of Food Intake

**DOI:** 10.1371/journal.pone.0032511

**Published:** 2012-03-08

**Authors:** Xiaoning Chen, Kara J. Margolis, Michael D. Gershon, Gary J. Schwartz, Ji Y. Sze

**Affiliations:** 1 Department of Molecular Pharmacology, Albert Einstein College of Medicine, Bronx, New York, United States of America; 2 Department of Medicine and Neuroscience, Albert Einstein College of Medicine, Bronx, New York, United States of America; 3 Department of Pathology and Cell Biology, Columbia University, New York, New York, United States of America; Universita Magna-Graecia di Catanzaro, Italy

## Abstract

Serotonin reuptake transporter (SERT) is a key regulator of serotonin neurotransmission and a major target of antidepressants. Antidepressants, such as selectively serotonin reuptake inhibitors (SSRIs), that block SERT function are known to affect food intake and body weight. Here, we provide genetic evidence that food intake and metabolism are regulated by separable mechanisms of SERT function. SERT-deficient mice ate less during both normal diet and high fat diet feeding. The reduced food intake was accompanied with markedly elevated plasma leptin levels. Despite reduced food intake, SERT-deficient mice exhibited glucose intolerance and insulin resistance, and progressively developed obesity and hepatic steatosis. Several lines of evidence indicate that the metabolic deficits of SERT-deficient mice are attributable to reduced insulin-sensitivity in peripheral tissues. First, SERT-deficient mice exhibited beta-cell hyperplasia and islet-mass expansion. Second, biochemical analyses revealed constitutively elevated JNK activity and diminished insulin-induced AKT activation in the liver of SERT-deficient mice. SERT-deficient mice exhibited hyper-JNK activity and hyperinsulinemia prior to the development of obesity. Third, enhancing AKT signaling by PTEN deficiency corrected glucose tolerance in SERT-deficient mice. These findings have potential implications for designing selective SERT drugs for weight control and the treatment of metabolic syndromes.

## Introduction

Disturbances in glucose homeostasis among psychiatric populations have been documented extensively [1], [2]. The mechanisms for this comorbidity are currently unknown, and are likely to involve diverse genetic, behavioral and environmental factors. Therefore, identifying the regulation of metabolic homeostasis by genes implicated in anxiety-related disorders may reveal insights into the pathophysiology of both mental and metabolic disorders. The serotonin reuptake transporter (SERT) is a major target of antidepressants. Selective serotonin reuptake inhibitors (SSRIs) are believed to exert antidepressant effects by blocking SERT uptaking serotonin (5-HT) from extracellular space, thereby enhancing 5-HT signaling [3]. In this study, we focused on the impact of constitutive SERT deficiency on energy balance and glucose homeostasis in mice.

Pharmacological studies have long implicated 5-HT in reducing food intake and body weight [4], [5], [6]. Subsequent genetic analyses in rodents demonstrated that hypothalamic 5-HT signaling controls food intake. For example, the 5-HT receptor 5HTR2C is highly expressed in the hypothalamus and 5HTR2C knockout mice develop hyperphagia, obesity and insulin resistance [7], [8]. In addition, the 5-HT receptor 5-HTR1B modulates the activity of melanocortin neurons to influence food intake [9].

By contrast, the impact of SSRIs on metabolism is less clear. Meta-analyses of clinical trials reported significant weight loss in subjects treated with fluoxetine [10], [11]. The effect of SSRIs on weight loss is, however, short term, and individuals regain or increase weight, despite continued SSRIs treatments [12], [13]. In some cases, SSRI treatments yielded hyperglycemia and a trend towards diabetes [13], [14], [15]. Accumulating evidence suggests that the effects of SSRIs on metabolisms are not merely secondary to improvement in affective states, but that 5-HT may act at both the brain and peripheral tissues to influence metabolism [16], [17], [18], [19]. However, the impact of SERT inactivation on glucose homeostasis and insulin signaling has not been systematically analyzed to date.

The purpose of this study was to test whether mice lacking SERT gene *Slc6a4* function (SERT−/−) exhibit reduced food intake, thereby protecting them against obesity and diabetes. To circumvent potential complications of reproductive hormone cycles in females, we focused on male mice. As expected, we found SERT−/− mice ate less. However, contrary to our expectations, SERT deficient mice exhibited glucose intolerance and insulin resistance, and progressively developed obesity and liver steatosis. We found that SERT−/− mice were hyperleptinemic, hyperglycemic and hyperinsulinemic prior to exhibiting a measurable increase in body fat content. We identified that C-Jun-N-terminal kinase (JNK) activity in the liver was constitutively elevated in SERT-deficient mice, whereas insulin-induced serine/threonine kinase AKT activation in the liver was attenuated. Based on these results, we propose that feeding and metabolism are regulated by separable mechanisms of SERT function and that SERT deficiency impairs the regulation of insulin signaling in peripheral tissues.

## Results

### SERT is distributed in multiple central and peripheral sites implicated in the control of metabolism

**Figure 1:**
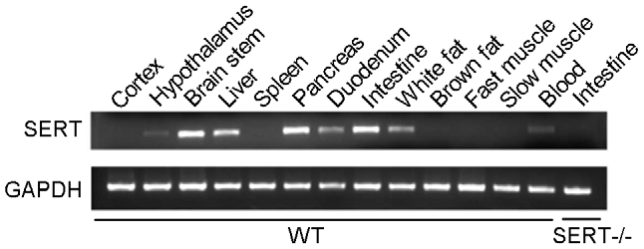
SERT mRNA detected by RT-PCR from WT mice tissues. Intestine tissue from a SERT−/− mouse is presented as a negative control. 6-month old mice were analyzed.

To begin to identify the putative role for SERT function in metabolism, we analyzed the distribution of SERT mRNA in C57BL/6J (WT) mice and mice bearing a targeted deletion in the SERT gene *Slc6a4* (SERT−/−) [20] by RT-PCR. Consistent with previous studies [21], [22], [23], we detected SERT mRNA in the brainstem, hypothalamus, as well as in blood, white adipose tissue, intestine, liver and pancreas (Figure 1). To validate the specificity of the probes, we analyzed SERT−/− mice. SERT mRNA was not expressed in any of those tissues in SERT−/− mice (Figure 1). These data support the idea that SERT may contribute to the control of metabolism in multiple central and peripheral tissues.

### SERT-deficient mice are hypophagic and hyperleptinemic

**Figure 2:**
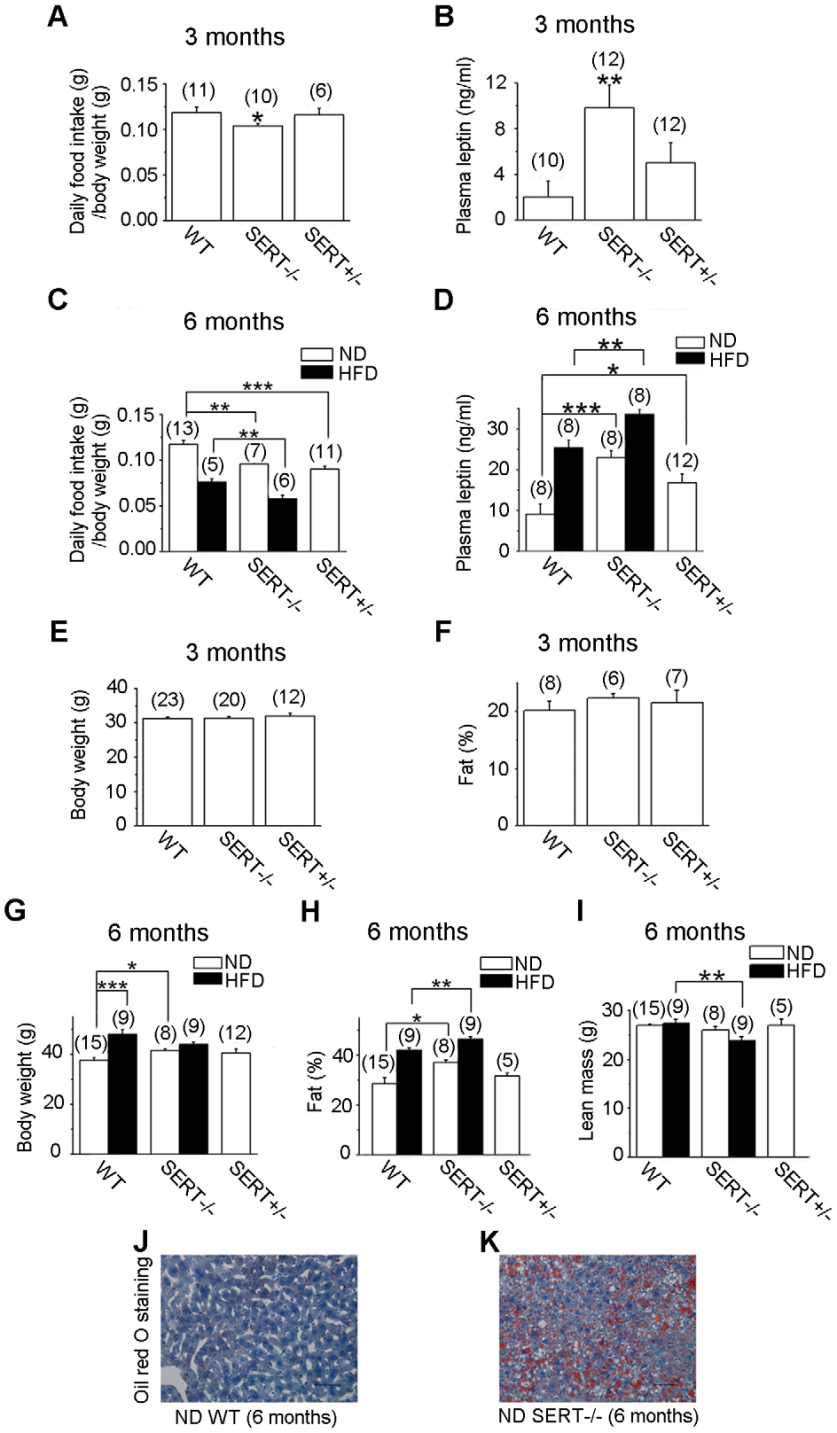
Characterization of food intake and adiposity of SERT-deficient mice. **A and C. Average daily food intake of 3- and 6-month old mice**. SERT mutant mice exhibited significantly reduced food intake during ND and HFD feeding, compared to corresponding WT mice. **B and D. Quantification of plasma leptin levels in 16 h fasted mice**. SERT mutant mice at the age of 3- and 6-month exhibited higher fasting lepin levels, as compared to corresponding WT controls. **E. Body weight at age of 3 months**. The differences among WT, SERT−/− and SERT+/− mice were not statistically significant. **F. Whole-body fat content at age of 3 months**. The differences among WT, SERT−/− and SERT+/− mice were not statistically significant. For example, p = 0.34 for the difference between WT and SERT−/− mice, Student's t-test. **G. Body weight at age of 6 months**. ND-fed SERT−/− mice exhibited higher body weight compared to ND-fed WT. The difference between SERT+/− and WT was, however, not statistically significant. HFD-fed WT mice exhibited higher body weight compared to ND-fed WT mice. However, the difference between HFD- and ND-fed SERT−/− mice was not statistically significant (p = 0.06, Student's t-test). **H. Whole-body fat content at age of 6 months**. Fat content was increased in SERT−/− mice fed ND and HFD compared to corresponding WT mice. **I. Absolute lean mass at age of 6 months**. Lean mass was reduced in HFD-fed SERT−/− mice, as compared to HFD-fed WT mice. Data in panels A–I are presented as means ± SEM. *p<0.05, **p<0.01, ***p<0.001, Student's t-test. The number of animals analyzed is indicated in parentheses. **J and K. Lipid content detected by Oil Red O staining of liver sections of WT and SERT−/− mice**. Scale bar, 50 µm.

In light of the well-established inhibitory effect of SSRIs on food intake, we monitored daily food consumption by WT and SERT-deficient mice. The amount of food consumed adjusted for body weight by SERT homozygous (SERT−/−) and heterozygous (SERT+/−) mutant mice was reduced compared to age-matched WT mice (Figure 2A, C; Figure S1) during normal diet (ND) feeding. We further analyzed food intake of SERT−/− mice during high fat diet (HFD) feeding, and found that daily intake of the HFD by SERT−/− was also reduced (Figure 2C). The levels of plasma triglycerides in SERT−/− and SERT+/− mice were indistinguishable from that in WT mice during ND feeding. HFD-fed SERT−/− mice exhibited lower plasma triglyceride levels compared to HFD-fed WT mice (Figure S2). Because SERT−/− mice showed a marked increase in extracellular 5-HT in the brain [24], these results could imply that SERT deficiency results in a greater hypothalamic 5-HT signaling leading to suppression of food intake.

In addition to 5-HT, the adipocyte-secreted peptide leptin inhibits food intake [25]. To assess the relationship between leptin and reduced food intake in SERT-deficient mice, we measured plasma leptin in SERT-deficient mice fasted for 16 hr. We found that fasting leptin levels were elevated in both SERT−/− and SERT+/− mice fed ND (Figure 2B, D). Fasting leptin levels were further increased in SERT−/− mice fed HFD (Figure 2D). These data suggest that elevated leptin levels could be a contributing factor for the hypophagia of SERT-deficient mice.

### SERT-deficient mice progressively develop obesity and liver steatosis but do not substantially increase body weight

Contrary to our expectations, we found that SERT-deficient mice developed age-dependent obesity, despite reduced food intake. At 3 months of age, the body weight and body fat content of WT and SERT−/− mice were similar (Figure 2E, F). At 6-months old, SERT−/− mice fed ND exhibited higher body weight and higher fat content compared to WT controls (Figure 2G, H). HFD feeding resulted in a further increase in the adiposity (Figure 2H). However, HFD-fed SERT−/− mice did not substantially further increase body weight compared to ND-fed SERT−/− mice, but they had reduced lean mass (Figure 2G, I). These data suggest that SERT deficiency promotes nutrient partitioning towards fat rather than simply causing an overall increase in body mass.

Previous studies indicated that major depressive and generalized anxiety disorders are over-represented in patients with nonalcoholic hepatic steatosis and such comorbidity is associated with obesity and type II diabetes [26], [27]. Consequently, the increased adiposity in SERT−/− mice prompted us to examine their hepatic fat content. Oil Red O staining of the liver from 3-month old SERT−/− mice did not show an appreciable increase in fat content (not shown). By contrast, we observed pronounced hepatic steatosis in 6-month old SERT−/− mice fed ND (Figure 2K). Collectively, these results show that SERT deficiency can lead to obesity and related metabolic disorders, even though it causes a reduction in food intake.

### SERT mutant mice exhibit glucose intolerance and insulin resistance prior to the development of obesity

**Figure 3:**
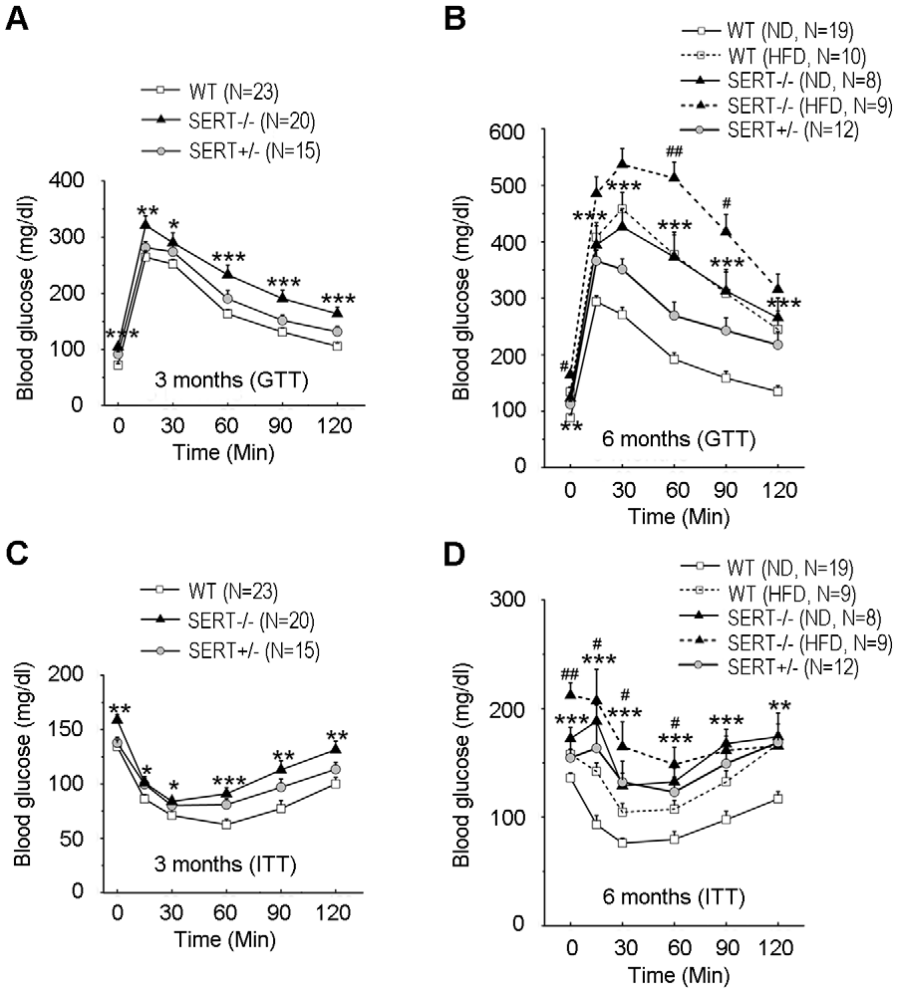
Characterization of glucose homeostasis in SERT-deficient mice. **A.** GTT (i.p., 1 g/kg) of 3-month-old mice after 16 h fast. **B.** GTT (i.p., 1 g/kg) of 6-month-old mice after 16 h fast. **C.** ITT (i.p., 1 U/kg) of 3-month-old mice after 6 h fast. **D.** ITT (i.p., 1 U/kg) of 6-month-old mice after 6 h fast. N, number of mice analyzed for each genotype. Data represent mean ± SEM. Student's t-test was used to analyze statistical significance of the difference between the following groups: SERT−/− vs. WT mice on ND feeding, *p<0.05, **p<0.01, ***p<0.001; SERT−/− vs. WT mice on HFD feeding, #p<0.05, ##p<0.01, Student's t-test. At 3 months of age, the differences in GTT of SERT+/− vs. WT mice were not statistically significant; however, there were significant differences between SERT+/− vs. WT mice in ITT. At 6 months of age, both SERT−/− and SERT+/− mice differed significantly from WT mice in GTT and ITT.

Since obesity is frequently associated with insulin resistance and type II diabetes, we wished to determine whether the increased adiposity in SERT-deficient mice is associated with altered glucose homeostasis. We found that fasting glucose levels were elevated in SERT−/− mice at 3- and 6-month of age (Figure 3A, B). Glucose tolerance tests (GTT) indicated that glucose clearance following injection of a bolus of glucose was affected in 3- and 6-month old SERT−/− mice (Figure 3A, B). Thus, SERT-deficient mice are hyperglycemic and have reduced glucose tolerance.

Glucose intolerance could reflect reduced insulin sensitivity of peripheral tissues in SERT-deficient mice. To test this possibility, we performed insulin tolerance tests (ITT). Figure 3C and D show that insulin-induced clearance of blood glucose was compromised in 3- and 6-month old SERT−/− mice fed ND. SERT+/− mice also exhibited glucose intolerance and insulin intolerance, albeit the deficits at the age of 3 months were modest (Figure 3), showing that even a partial SERT deficiency may impair glucose homeostasis and insulin responses.

To examine the metabolic response to increased dietary fat in SERT-deficient mice, 3-month old mice were fed with a HFD for three months, and their glucose tolerance and insulin sensitivity were analyzed by GTT and ITT, respectively. Both WT and SERT−/− mice fed the HFD developed significant glucose intolerance (Figure 3B) and insulin resistance (Figure 3D) compared to their ND-fed controls. HFD-fed SERT−/− mice exhibited higher fasting glucose levels, and higher glucose levels at 60 min and 90 min post-injection of a bolus of glucose during GTT, compared to HFD-fed WT mice (Figure 3B). HFD-fed SERT−/− mice also exhibited greater insulin resistance than HFD-fed WT mice during ITT (Figure 3D). Remarkably, 3-month old SERT−/− mice did not exhibit an increase in body weight and body fat content (Figure 2E, F) but they did manifest glucose and insulin intolerance, suggesting that the metabolic deficits develop prior to a significant increase in the adiposity.

### SERT-deficient mice exhibit hyperinsulinemia and hypertrophic pancreatic islets

**Figure 4:**
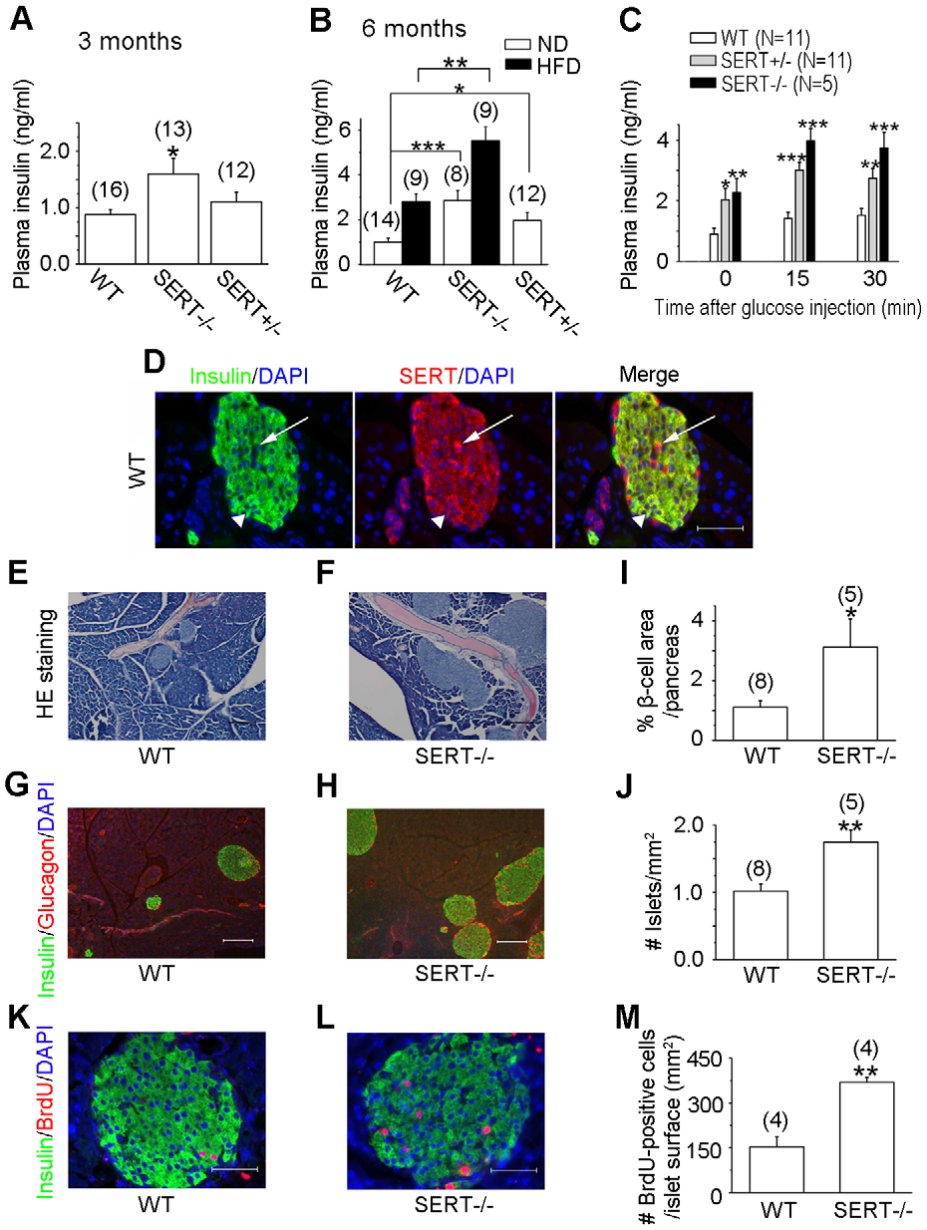
Biochemical, histological and immunohistochemical analyses of insulin secretion and pancreatic beta-cell morphology in SERT-deficient mice. **A and B. Quantification of basal levels of plasma insulin in 16 h fasted mice at the age of 3 months (A) and 6 months (B).** SERT mutant mice exhibited elevated fasting insulin levels during ND and HFD feeding as compared to corresponding WT control mice, *p<0.05, **p<0.01, ***p<0.001, Student's t-test. The number of mice analyzed is indicated in parentheses. **C. Quantification of glucose-induced plasma insulin levels**. 6-month old mice fasted for 16 h were injected with glucose (i.p., 1 g/kg). Insulin levels in plasma samples collected before and 15 and 30 min post glucose administration were analyzed. SERT−/− and SERT+/− mice exhibited higher insulin levels at all tested time points, compared to corresponding WT mice. Data represent mean ± SEM. *p<0.05, **p<0.01, ***p<0.001, Student's t-test. N, number of animal analyzed. **D. Immunofluorescent staining of pancreata of WT mice**. The left panel shows insulin (green), the middle panel shows SERT (red) co-stained with DAPI (blue), and the right panel shows merge of the fluorescence. Noticing SERT staining in both insulin-expressing cells (as pointed by a triangle) and non-insulin cells (as pointed by an arrow) in the islets. Scale bar, 50 µm. **E and F.** H&E staining of pancreatic tissue sections from WT and SERT−/− mice. Noticing enlarged islet mass in the SERT−/− background. Scale bar, 100 µm. **G and H.** Triple-staining of pancreas of WT and SERT−/− mice for insulin (green), glucagon (red) and DAPI (blue). Scale bar, 200 µm. **I and J.** Quantification of islet mass in WT and SERT−/− mice. **I.** Beta-cell density was determined by calculating the percentage of insulin-labeled area on serial sections of pancreata. **J.** Islet density was determined by calculating the average number of islets on the pancreatic sections. The value from SERT−/− mice was compared with that of WT mice, *p<0.05, **p<0.01, Student's t-test. The number of mice analyzed is indicated in parentheses. **K and L.** Representative photomicrographs showing BrdU incorporation (red) in insulin-labeled cells (green) in pancreatic tissue sections of WT and SERT−/− mice. Scale bar, 50 µm. **M.** Beta-cell proliferation was estimated by calculating the number of beta cells incorporated BrdU relative to the islet area. Data represent mean ± SEM, **P<0.01, Student's t-test. The number of animals analyzes is indicated in parentheses.

The poor glucose handling in SERT-deficient mice could be completely ascribed to impaired insulin sensitivity. Alternatively, SERT deficiency could result in reduced pancreatic beta-cell secretion of insulin. To test the second possibility, we analyzed insulin secretion in SERT-deficient mice. We found that fasting plasma insulin levels were elevated in both SERT−/− and SERT+/− mice at the age of 3 and 6 months kept on ND, although the increase in 3-month old SERT+/− mice was not statistically significant (Figure 4A, B). To evaluate the impact of SERT deficiency on glucose-induced insulin secretion, we measured plasma insulin levels following bolus injection of glucose. Glucose induced insulin in both SERT-deficient and WT mice as measured at 15 min and 30 min post glucose injection (Figure 4C). While the absolute values of plasma insulin in SERT−/− and SERT+/− mice were higher than that in WT mice, the fold-increase of insulin was similar among tested strains. Thus, SERT deficiency does not attenuate insulin secretion, but it causes hyperinsulinemia.

Hyperinsulinemia frequently reflects an adaptive pancreatic response to increased metabolic demand. In the state of chronically increased nutrient load, obesity, and insulin resistance, pancreatic beta cells increase insulin secretion by expanding beta-cell mass [28]. Indeed, following three months of HFD feeding, fasting plasma insulin levels were increased in WT as well as in SERT−/− mice, compared to their ND-fed controls, with the absolute values of plasma insulin in SERT-deficient mice higher (Figure 4B). To begin to understand the impact of SERT deficiency on pancreatic function, we carried out histological analyses of islets. Consistent with the abundant SERT mRNA in the pancreas (Figure 1), immunohistochemical staining of pancreatic tissues from 6-month old WT mice showed high levels of SERT in non-insulin labeled cells of islets and lower SERT levels in insulin labeled cells (Figure 4D). Histological analysis of hematoxylin and eosin (H&E) stained pancreatic tissue sections of 6-month old mice showed that the overall structure and organization were preserved in SERT−/− mice (Figure 4E, F). Immunostaining for insulin and glucagon indicated normal distributions of islet alpha- and beta-cells (Figure 4G, H). However, we found that the size and number of islets both were increased in SERT−/− mice. On average, SERT−/− mice exhibited 70% more pancreatic islets and a total of a 3-fold increase in beta-cell mass compare to WT mice (Figure 4I, J).

To determine the mechanism underlying islet hypertrophy of SERT−/− mice, we assessed beta-cell proliferation and apoptosis in SERT−/− and WT mice. Judged by BrdU incorporation of beta cells, there was an approximately 2-fold increase in beta-cell proliferation in SERT−/− mice (Figure 4K–M). In contrast, SERT−/− and WT mice showed a similar frequency of apoptosis of the beta cells, as judged by TUNEL staining (Figure S3). Collectively, these results suggest that glucose intolerance of SERT mutant mice is more likely due to reduced response to insulin, rather than reduced pancreatic insulin secretion.

### Insulin-induced AKT activation is blunted in SERT mutant liver and muscle

**Figure 5:**
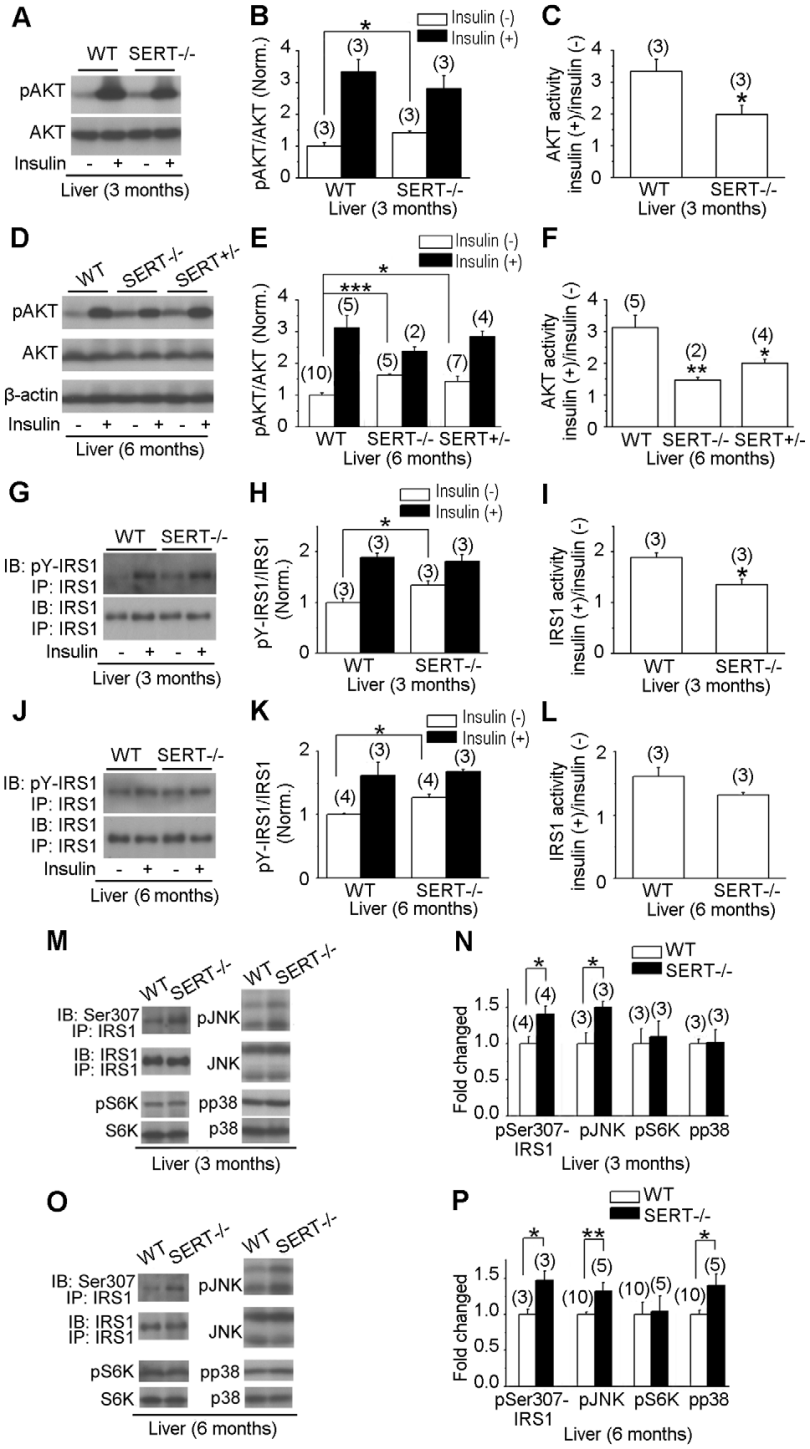
Western blot analysis of insulin signaling component activity in SERT-deficient mice. **A–F.** AKT activity in the liver of 3- and 6-month old WT and SERT-deficient mice was evaluated by western blot analysis of AKT Ser473 phosphorylation (pAKT) before and after insulin injection. **G–L.** IRS1 activity in the liver of 3- and 6-month old WT and SERT-deficient mice was evaluated by western blot analysis of tyrosine-phosphorylated IRS1. IRS1 was first immunoprecipitated with anti-IRS1 antibody (IP-IRS1) and then blotted with anti-IRS1 (IB-IRS1) or anti-phosphotyrosine antibodies (IB-pY-IRS1). **A, D, G and J** show images of representative western blot results. **B and E** show densitometric quantification of the ratio of pAKT vs. total AKT, and **H and K** show densitometric quantification of the ratio of pY-IRS1 vs. total IRS1. Insulin (−), basal activity determined from the tissues collected from16 h fasted mice. Insulin (+), insulin-induced activity determined from the tissues collected from mice 20 min post insulin injection. The value of WT treated with insulin and mutants with and without insulin treatment is normalized to that of WT without insulin treatment. **C and F** show the ratio of pAKT before and after insulin injection for each genotype. **I and L** show the ratio of pY-IRS1 before and after insulin injection. The basal pAKT and pY-IRS1 were elevated, but the net increase in pAKT and pY-IRS1 following insulin injection was attenuated in SERT mutant mice. **M–P.** Western blot analysis of phospho-Ser307-IRS1, phospho-JNK (pJNK), phospho-S6K (pS6K), and phospho-p38 MAPK (pp38) in the liver of 16 h fasted 3- and 6-month old SERT mutant mice. **M and O** show images of representative western blot results. **N and P** show densitometric quantification of pSer307-IRS1, pJNK, pS6K and pp38. The amounts of phosphorylated proteins are normalized to that of total corresponding proteins. Shown are the relative values of SERT−/− mice to WT, with the average value from WT mice defined as 1. Data represent mean ± SEM, *p<0.05, **p<0.01, Student's t-test. The number of mice analyzed is indicated in parentheses.

To investigate the molecular mechanisms underlying the insulin resistance of SERT-deficient mice, we analyzed insulin signaling in the liver of 3- and 6-month old mice. The serine/threonine kinase AKT signaling is critical for PI3K-mediated insulin action on glucose uptake and storage in liver and muscle [29]. While AKT2 deficiency impairs insulin action on liver metabolism [30], [31], elevated basal AKT activity contributes to insulin resistance in obese mice induced by HFD [32] and elevated basal PI3K signaling is associated with insulin resistance in skeletal muscle and glucose intolerance in men [33]. Indeed, fasting AKT activity was significantly elevated in the liver of SERT−/− and SERT+/− mice compared to WT mice, as judged by the level of phosphorylated AKT (pAKT) using western blot analyses (Figure 5A, B, D, E). Importantly, insulin-induced increase of pAKT was attenuated in SERT−/− and SERT+/− mice, although the absolute amounts of pAKT were similar between WT and the SERT mutants following insulin injection (Figure 5B, C, E, F), again, consistent with that seen in glucose intolerant patients [33]. Fasting AKT activity was also elevated while insulin-induced increase of pAKT was attenuated in the muscle of SERT−/− and SERT+/− mice (Figure S4).

Insulin-receptor substrate (IRS) proteins are cytoplasmic adaptor proteins that couple insulin signals to PI3K activity [29]. Consistent with the elevated basal AKT activity, the basal activity of IRS1 was elevated in the liver of 3- and 6-month old SERT−/− mice compared to WT mice, as judged by the level of tyrosine phosphorylated IRS1 (pY-IRS1) using western blot analyses (Figure 5G, H, J, K). Further, insulin-induced increase of pY-IRS1 was attenuated in SERT−/− mice (Figure 5H, I, K, L), parallel to the reduced insulin-induced AKT activation. Collectively, these data suggest that insulin stimulation of PI3K signaling in the liver and muscle is diminished in SERT-deficient mice. 3-month old SERT−/− mice did not display obesity (Figure 2E, F) and hepatic steatosis (data not shown), but their IRS1 and AKT responses to insulin stimulation were reduced, further indicating that SERT−/− mice develop insulin resistance prior to a significant increase in the adiposity.

### JNK activity was increased in SERT mutants

Stress-induced kinases, JNK, S6 kinase (S6K) and p38 MAP kinase, have been shown to inhibit insulin activation of the PI3K pathway [29], [34]. The activities of these kinases are regulated by distinct metabolic and physiological stress, and chronic activation of JNK, S6K and p38 is found to promote glucose intolerance and insulin resistance [34], [35], [36]. To evaluate the activities of those stress kinases prior to and after the development of obesity in SERT deficient mice, we analyzed JNK, S6K, and p38 activities in the liver of 3- and 6-month old mice, using western blot analysis. At 3-month old, WT and SERT−/− mice exhibited similar fasting S6K and p38 activities, while fasting JNK activity was significantly increased in the SERT−/− mice, as judged by the levels of phosphorylated S6K (pS6K), p38 (pp38) and JNK (pJNK) (Figure 5M, N). At 6-month old, fasting levels of both pJNK and pp38 were increased in SERT−/− mice, while pS6K levels were not significantly changed (Figure 5O, P). These data suggest that the basal JNK activity was increased in SERT-deficient mice prior to the development of obesity, while the basal p38 activity was increased in parallel with the increase in the adiposity.

JNK activation is thought to inhibit insulin receptor signal transduction by phosphorylating Ser307 of IRS1 [37], [38]. To test JNK activity directly, we determined the basal level of pSer307-IRS1 in the liver of SERT−/− and WT mice. Figure 5M–P show that the basal pSer307-IRS1 levels were significantly elevated in 3- and 6-month old SERT−/− mice compared to WT controls. These results are consistent with the idea that a constitutive increase in JNK signaling contributes to insulin resistance in SERT deficient mice.

### Glucose intolerance of SERT-deficient mice can be partially corrected by reducing PTEN activity

**Figure 6:**
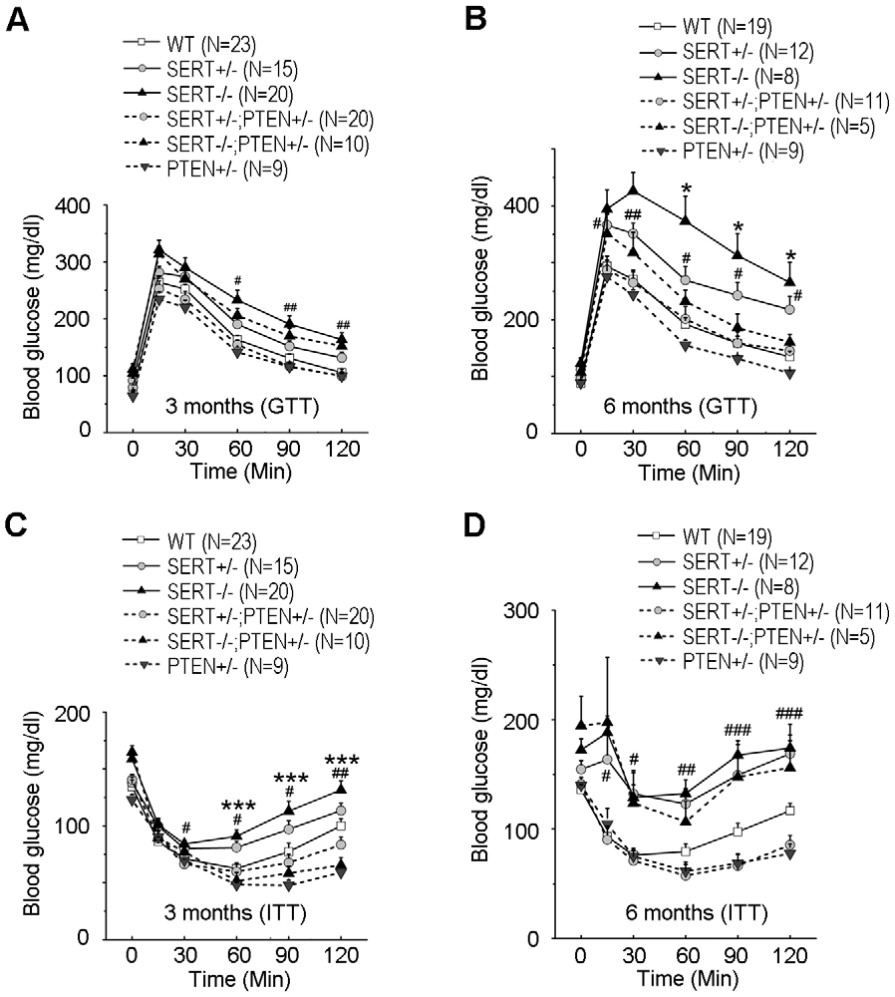
Reduced PTEN function corrected glucose tolerance in SERT mutants. **A and B** show GTT (i.p., 1 g/kg), and **C and D** show ITT (i.p., 1 U/kg). The SERT−/−; PTEN+/− mice exhibited improved insulin sensitivity at 3 months of age and improved glucose tolerance at 6 months of age compared to age-matched SERT−/− mice, *p<0.05, ***p<0.001. The SERT+/−; PTEN+/− mice exhibited significantly improved glucose tolerance and insulin sensitivity at the age of 3- and 6-months, compared to age-matched SERT+/−, #p<0.05, ##p<0.01, ###p<0.001, Student's t-test. N, number of mice analyzed.

We next asked whether a genetically induced increase in AKT signaling could restore glucose tolerance of SERT mutant mice. The PTEN phosphatase is a negative regulator of PI3K signaling pathway [39]. PTEN-null mice are lethal but conditional PTEN deletion and PTEN+/− mice have elevated AKT signaling [39]. We generated SERT−/−; PTEN+/− and SERT+/−; PTEN+/− double mutant mice and analyzed their glucose tolerance and insulin sensitivity using GTT and ITT. Consistent with previous reports [40], [41], PTEN+/− mice exhibited greater glucose tolerance and insulin sensitivity than WT mice (Figure 6). Remarkably, the PTEN+/− mutation fully corrected glucose tolerance during GTT and insulin-induced glucose clearance during ITT in SERT+/− mice (Figure 6). Glucose tolerance and insulin sensitivity of the SERT−/−; PTEN+/− double mutants were also improved, compare to SERT−/− mice (Figure 6). One potential explanation for these observations is that elevated basal AKT and JNK activities desensitize the response to insulin, leading to glucose intolerance, and that reduced PTEN function boosts AKT signaling thus improving glucose handling. However, PTEN+/− only partially suppressed the metabolic symptoms in SERT−/− mice, suggesting additional effects of SERT deficiency on metabolic homeostasis.

**Figure 7:**
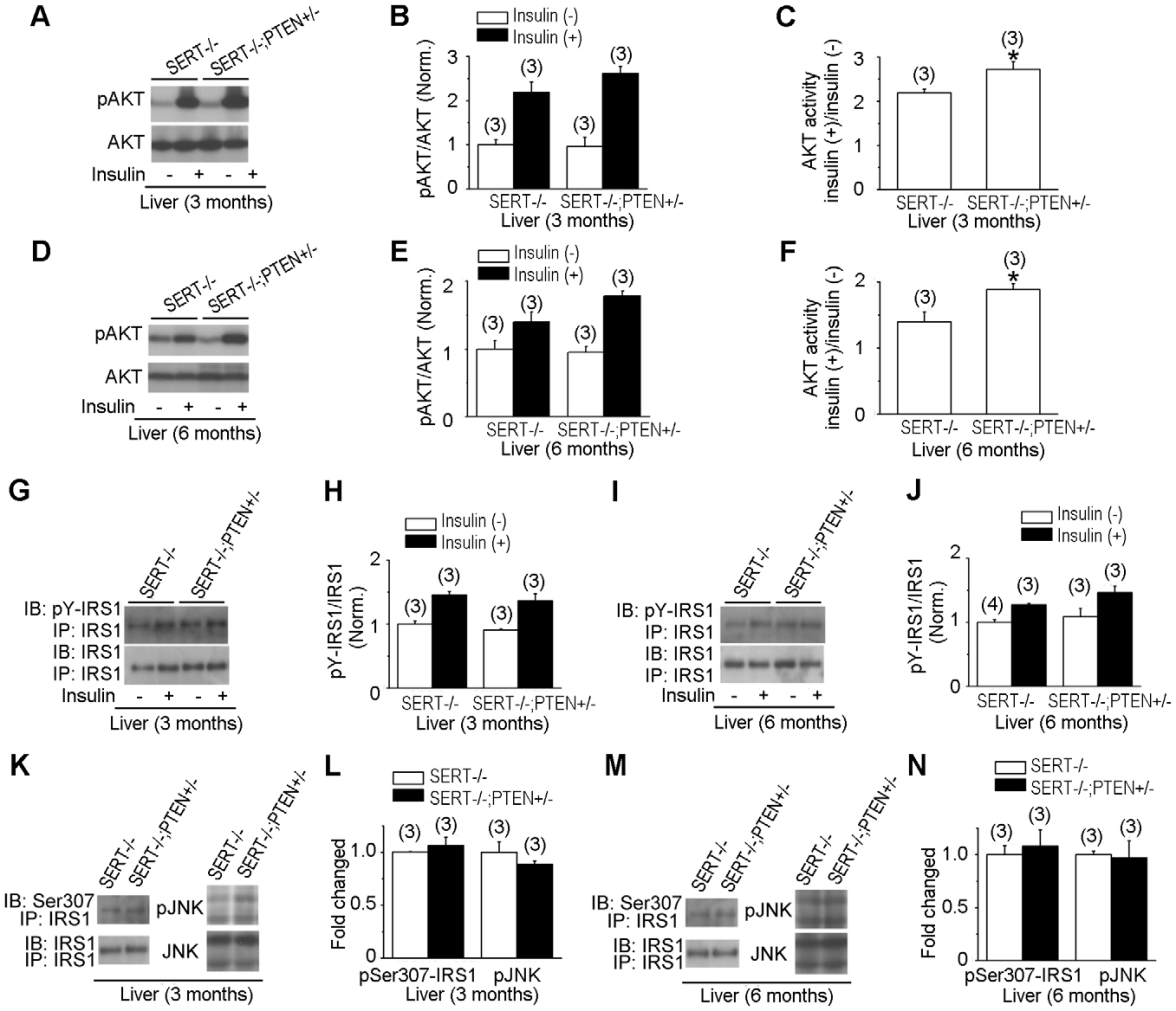
Western blot analysis of insulin signaling component activity in SERT−/−;PTEN+/− mice. **A–F.** AKT activity in the liver of 3- and 6-month old SERT−/−; PTEN+/− and SERT−/− mice was evaluated by AKT Ser473 phosphorylation (pAKT) before and after insulin injection. **G–J.** IRS1 activities in the liver of 3- and 6-month old SERT−/−;PTEN+/− and SERT−/− mice were evaluated by tyrosine-phosphorylated IRS1. IRS1 was first immunoprecipitated with anti-IRS1 antibody (IP-IRS1) and then blotted with anti-IRS1 (IB-IRS1) or anti-phosphotyrosine antibodies (IB-pY-IRS1). **A, D, G and I** show images of representative western blot results. **B and E** show densitometric quantification of the ratio of pAKT vs. total AKT, and **H and J** show densitometric quantification of the ratio of pY-IRS1 vs. total IRS1. Insulin (−), basal activity determined from the tissues collected from16 h fasted mice. Insulin (+), insulin-induced activity determined from the tissues collected from mice 20 min post insulin injection. The value of WT treated with insulin and mutants with and without insulin treatment is normalized to that of WT without insulin treatment. **C and F** show the ratio of pAKT before and after insulin injection for each genotype. The net increase in pAKT amounts following insulin injection was elevated in SERT−/−; PTEN+/− mice relative to SERT−/− mice, *p<0.05, Student's t-test. **K–N.** Western blot analysis of phospho-JNK (pJNK) and phospho-Ser307-IRS1 in the liver of 16 h fasted 3- and 6-month old SERT−/−; PTEN+/− and SERT−/− mice. **K and M** show images of representative western blot results. **L and N** show densitometric quantification of pJNK and pSer307-IRS1. The amounts of phosphorylated proteins were normalized to that of total corresponding proteins. Shown are the relative values of SERT−/−; PTEN+/− mice to SERT−/− mice, with the average value from SERT−/− mice defined as 1. Data represent mean ± SEM. The number of mice analyzed is indicated in parentheses.

To validate that PTEN+/− indeed enhanced AKT signaling in SERT−/− background, we compared pAKT levels in the liver of SERT−/−; PTEN+/− double mutant mice with that in SERT−/− mice. As shown in Figure 7A–F, insulin-induced phosphorylation of AKT was increased in the liver of 3- and 6-month old SERT−/−; PTEN+/− mice compared to that of SERT−/− mice. As a control, we analyzed insulin-induced phosphorylation of IRS1, an upstream regulator of PI3K. Figure 7G–J show that pY-IRS1 levels in the liver of SERT−/−; PTEN+/− mice were similar to that in SERT−/− mice. These data suggest that reduced PTEN phosphatase activity results in an increase in pAKT, thereby enhancing AKT downstream pathways, but it does not correct IRS1 response to insulin. Consistent with this idea, basal JNK activity in SERT−/−; PTEN+/− mice was as high as in SERT−/− mice, based on western blot analyses of pJNK and p-Ser307-IRS1 in the liver (Figure 7K–N).

## Discussion

The data presented here show that SERT-deficient mice progressively develop obesity and hepatic steatosis, despite reduced food intake. We also show that SERT-deficient mice exhibit hyperglycemia and insulin resistance, both of which are characteristic features of diabetes. Our data suggest that glucose intolerance and insulin resistance of SERT-deficient mice are attributable, at least in part, to impaired PI3K and JNK signaling in the peripheral tissues. These findings suggest that food intake and glucose homeostasis may be regulated by separable 5-HT signaling pathways in multiple peripheral organs and are influenced by SERT function. The metabolic defects of SERT+/− mice indicate that even a partial SERT deficiency can profoundly impair glucose homeostasis and insulin sensitivities.

### Impact of SERT function on metabolic homeostasis is multifactorial

Our analyses of SERT-deficient mice revealed a new effect of SERT function on food intake. We found that basal levels of leptin, the major inhibitory hormone of food intake [25], were markedly increased in SERT−/− and SERT+/− mice. Since SERT deficiency presumably results in increased 5-HT signals [24], excessive 5-HT signaling may inhibit food intake via a mechanism enhancing leptin secretion. Both SERT deficiency and leptin have been shown to inhibit bone mass accrual [42], [43]. Consequently, it is plausible that elevated circulatory leptin underscores the effects of SERT deficiency not only on feeding, but also on bone mass and perhaps other aspects of hypothalamic function.

One interesting finding uncovered from our study is that SERT-deficient mice develop glucose intolerance, insulin resistance and obesity, despite reduced feeding. SERT deficiency has been associated with anxiety [18] and glucocorticoids play an important role in insulin sensitivity [44]. However, basal plasma corticosterone was not significantly altered in SERT-deficient mice (Figure S5), consistent with a previous report [45]. It has been reported that SERT−/− mice were hypoactive during exploratory and social behavioral tests [46], although another study indicated that SERT−/− rats did not reduce total distance traveled during behavioral assays [47]. Thus, hypoactivity could be a contributing factor to obesity of SERT-deficient mice. However, our studies of 3- and 6-month old SERT-deficient mice suggest that glucose intolerance and insulin resistance manifest prior to an appreciable increase in the adiposity. Previously, it was reported that in *C. elegans* 5-HT regulates feeding and fat metabolism through independent molecular mechanisms [48]. Thus, 5-HT is likely to regulate distinct targets to control the perception of satiety and energy metabolism of animals, and the dual action of 5-HT may represent an evolutionary ancient strategy for metabolic homeostasis conserved in disparate organisms.

### The impact of SERT deficiency on insulin signaling in the peripheral tissues

Several lines of experimental results suggest that SERT-deficiency impairs insulin action on peripheral tissues. First, basal pAKT and pY-IRS1 levels were elevated, while insulin-induced increase of pAKT and pY-IRS1 was attenuated in the liver of SERT-deficient mice. Increased basal pAKT is considered as a trigger of insulin resistance by desensitizing insulin signaling [32]. Second, PTEN+/− mutation enhanced AKT signaling in SERT-deficient mice and improved their glucose tolerance. Third, SERT−/− mice constitutively increase JNK activity in the liver, coupled with enhanced the phosphorylation at the inhibitory sites of IRS1, a key regulator of IR signaling. It is interesting to note that the metabolic deficits in SERT-deficient mice, including glucose intolerance, insulin resistance, and increased JNK activity, all arise prior to a measurable increase in the adiposity. These data suggest that the metabolic deficits of SERT-deficient mice are not the consequence of obesity. Further studies are required to determine the mechanisms by which SERT function control glucose homeostasis and insulin sensitivity. Reduced SERT function has been implicated in anxiety-related disorders [18]. Low expression alleles of the human SERT gene are linked to post-traumatic syndrome and anxiety-related traits [49], [50]. SERT+/− mice exhibit elevated anxiety behavior [51]. We found that SERT+/− mice develop significant metabolic deficits, showing that a partial reduction in SERT function may also perturb metabolic homeostasis and confer a risk factor for diabetes.

## Materials and Methods

### Animals

All mice used in the experiments were bred on a C57BL/6 background and housed in facilities accredited by the American Association for Laboratory Animal Care (AALAC #30549) on a 12-h light/dark cycle with ad libitum access to normal diet (D5053; Research Diets) or a high-fat diet (D12492; Research Diets). The C57BL/6J strain (Jackson Laboratory) was used as a WT. Targeted deletion of the SERT gene *Slc6a4−/−* (SERT−/−) has been described previously [20]. PTEN+/− mice [52] were obtained from NCI. SERT−/−; PTEN+/− and SERT+/−; PTEN+/− double mutants were generated from this work. Mice were genotyped by PCR analyses of genomic DNA extracted from tail tissue. All studies were performed using male mice at the age of 3 and 6 months. The animal studies were approved by the Institute Animal Care and Use Committee (IACUC #20090508) of the Albert Einstein College of Medicine.

### SERT mRNA profiling

Total RNA was extracted from tissues of 6-month old WT mice using TRIZOL reagents (Invitrogen). 2 mg of total RNA from each tissue were used for reverse transcription with the Superscript III reverse transcriptase (Invitrogen), and the resultant cDNA was amplified by PCR with the following primers: SERT: 5′-CGCAGTTCCCAGTACAAGC-3′ and 5′-CGTGAAGGAGGAGATGAGGT-3′; GAPDH: 5′-GCCTTCCGTGTTCCTACCC-3′ and 5′-TGAAGTCGCAGGAGACAACC-3′.

### Food intake and body composition measurements

To evaluate food intake, food consumption by individually housed mouse was recorded daily for a period of one week or 20 days. Daily HFD intake was monitored using 6-month old mice that had fed on the HFD for 10 weeks. Briefly, a measured amount of ND or the NFD was provided daily to individually housed mouse, and leftover and spillover were collected and weighed on the following day, and the difference was calculated as daily net food intake of the mouse. While potential error attributable to loss of food particles over spillover cannot be excluded, the bedding was carefully screened to minimize potential error. Body weight of test mice was recorded every day, and the amount of food consumed by each mouse adjusted for its bodyweight was calculated as daily food intake. Data represent the average of daily food intake of mice analyzed in parallel for each genotype. Body composition of mice at indicated age was determined by nuclear magnetic resonance using an EchoMRI analyzer (Echo Medical Systems).

### Metabolic measurements

Metabolic parameters were measured in 3- and 6-month-old mice fasted for 16 h, unless specified otherwise. Glucose levels in the blood samples obtained by tail bleeding were measured using a glucometer (Bayer HealthCare). For measuring triglycerides, leptin and insulin levels, blood was collected from the tail vein and the plasma was isolated by centrifugation of the blood samples. Total plasma triglycerides were determined using the Triglyceride Determination Kit (Sigma-Aldrich). Plasma insulin and leptin levels were determined using the Rat/Mouse Insulin ELISA Kit (Millipore) and Mouse Leptin ELISA Kit (Crystal Chem), respectively. To measure insulin secretion in response to glucose, mice fasted for 16 h were injected with D-glucose (1 g/kg body weight), and blood was collected from the tail vein prior to the injection and at the indicated time points post injection, and the plasma insulin levels were determined.

Glucose tolerance tests (GTT) and insulin tolerance tests (ITT) were carried out using 3- and 6-month old mice. For GTT, 16 h fasted mice were injected intraperitoneally (i.p.) with D-glucose (1 g/kg body weight), and the blood glucose level was determined by tail bleeding immediately before and at indicated time points after glucose injection using the glucometer. ITT was performed on 6 h fasted mice by intraperitoneal injection of insulin (1 unit/kg body weight; Sigma-Aldrich), and the blood glucose concentrations before and at indicated time after insulin injection were determined by the glucometer. To examine diet-induced changes in glucose metabolism, 3-month old mice were fed ND or the HFD for 3 months, and GTT and ITT were performed. For measuring corticosterone, blood was collected from mice with food ad libitum, and the concentrations of corticosterone in the plasma was determined using Corticosterone Enzyme Immunoassay Kit (Enzo life science).

### Lipid staining of hepatic tissues

The liver was dissected from 3- and 6-month old mice and fixed in 4% paraformaldehyde solution overnight and placed in a 30% sucrose solution. Fixed hepatic tissues were embedded in OCT compound (Sakura Finetek) on dry ice, sectioned to slices at 9 µm thickness, and stained with Oil Red O.

### Histological and morphometric analysis of pancreatic tissues

Pancreatic tissues from 6-month old mice were fixed in 4% paraformaldehyde solution overnight, embedded in OCT compound and sectioned into serial slices at 9 µm thickness. Sections were stained with hematoxylin-eosin (H&E) for morphological evaluation. For immunohistochemical analyses, the following antibodies were used in indicated dilution: rabbit anti-SERT (1∶400; Chemicon International, Temecula, CA), guinea pig anti-human insulin (1∶400; Abcam), rabbit anti-glucagon (1∶500; gift of D. Cai), rat anti-BrdU (1∶400; ABcam). Fixed tissues were incubated with primary antibodies overnight in a humid chamber, and labeled by Alexa Fluor conjugated secondary antibodies (Molecular Probe) in 1∶800 dilution: Alexa Fluor 488 goat anti-guinea pig (for insulin), Alexa Fluor 594 goat anti-rabbit (for glucagon and SERT), and Alexa Fluor 594 goat anti-rat (for BrdU). Staining patterns were visualized under an AxioImager Z1 microscope equipped with proper filters, and images were captured using Axiocam MR digital camera (Zeiss).

For islet morphometric analyses, 4–5 sections (9 µm) separated by 250 mm from serial sections of individual pancreas were analyzed. The islet areas on each section were evaluated. Islet areas with clustered insulin-positive cell >850 µm^2^, which contains estimated 5 cells (∼170 µm^2^/β-cell) [53], were scored, using the Image-Pro Plus 5 Software (Media Cybernetics). The percentage of beta-cell area was calculated by dividing the value of analyzed insulin-positive areas on one section by the values of the total area of this section. The islets density was calculated by dividing the number of islets on one section by the total area of this section. The values from all analyzed sections of each pancreas were pooled.

Beta-cell proliferation was evaluated by 5-bromo-2′-deoxyuridine (BrdU) incorporation. 6-month old mice were injected intraperitoneally with 100 mg/kg/day of BrdU (Sigma-Aldrich) for three days and sacrificed afterwards. Pancreatic tissues were removed, fixed and embedded as described above and stained with insulin and BrdU. Islet cells stained by both BrdU and insulin were scored as BrdU-positive beta cells. For every pancreas, at least 20 islets from 3 sections were counted. The proliferation rate was calculated as the total number of BrdU-positive beta cells over the value of the total area of islets analyzed.

Apoptosis analysis was performed on pancreas sections from 6-month old mice by the terminal deoxynucleotidyltransferase-mediated dUTP-biotin nick end labeling (TUNEL) method using the In Situ Cell Death Detection Kit (Roche Applied Science).

### Immunoblot analysis

Hepatic tissues and muscle were isolated from mice at indicated age. The tissues were homogenized in lysis buffer (30 mM HEPES (pH 7.4), 150 mM NaCl, 10% glycerol, 1% Triton X-100, 5.0 mM EGTA, 1.0 mM phenylmethylsulfonyl fluoride, 3.0 µM aprotinin, 10 µM leupeptin, 5.0 µM pepstatin A, 25 mM benzamidine, 2 mM sodium vanadate, 5.0 mM glycerol phosphate, 100 mM NaF, 1.0 mM ammonium molybdate and 30 mM tetrasodium pyrophosphate). Following centrifugation at 14000 rpm for 20 minutes at 4°C, protein extracts were resolved by SDS-PAGE and transferred onto Polyvinylidene fluoride (PVDF) membrane. For IRS1 studies, IRS1 was first immunoprecipitated from liver lysate (2 mg) with anti-IRS1 antibody (Cell signaling Technology), and immunoprecipitates were washed three times with lysis buffer and used for western blotting. The following antibodies were used: anti-β-actin antibody, anti-phospho-Ser473 AKT antibody, anti-Akt antibody, anti-SAPK/JNK antibody, anti-phospho-Thr183/Tyr185-SAPK/JNK antibody (Cell Signaling Technology), anti-p70 S6 Kinase antibody and anti-p38 MAP Kinase antibody (gifts from C. Chow), anti-phospho-p70 S6Kinase (Thr389) antibody and anti-phospho-Thr180/Tyr182-p38 MAP Kinase antibody (gifts of S. Horwitz), anti-phosphotyrosine antibody and anti-pSer307-IRS1 antibody (Millipore). Protein levels were quantified by densitometry using Image-Pro software (Media Cybernetics). The value of pAKT, pJNK, pS6K, pp38, pY-IRS1 and pSer307-IRS1 was normalized respectively to that of total AKT, JNK, S6K, p38 and IRS1. To measure insulin-induced AKT and IRS1 activation, mice fasted for 16 h were injected with insulin (1 unit/kg body weight), and the liver and muscle were collected 20 min post-injection, and western blot analyses were performed.

### Statistical analysis

Data are presented as mean ± SEM. Statistical significance was evaluated using 2-tailed, unpaired Student's t-test. Differences were considered significant at P values of less than 0.05.

## Supporting Information

Figure S1 **Effects of SERT deficiency on food intake.** **A.** Daily food intake of 3-month old WT, SERT−/− and SERT+/− mice. Amount of food consumed by individually housed mice and the body weight of each mouse were monitored for 20 consecutive days. Data represent the average of daily food intake adjusted for body weight ± SEM. **B.** Summary of the entire monitoring period for each genotype. *, p<0.05 Student's t-test. N, number of mice analyzed.
(TIF)

Figure S2 **Quantification of plasma triglyceride levels in 6-month old mice fasted for 16 h.** The differences between SERT mutant mice and WT mice fed ND are not statistically significant (SERT−/−, p = 0.58; SERT+/−, p = 0.66). HFD-fed SERT−/− mice exhibited lower triglyceride levels than HFD-fed WT mice, *, p<0.05 Student's t-test. The number of mice analyzed is indicated in parentheses.
(TIF)

Figure S3 **Representative photomicrographs showing TUNEL staining (red) of sections of pancreata to visualize apoptotic cells.** There was no appreciable difference detected between WT and SERT−/− mice. All animals analyzed were 6-month old fed ND. Scale bar, 50 µm.
(TIF)

Figure S4 **Western blot analysis of AKT activity in the muscle of 6-month old WT and SERT-deficient mice.** AKT activity was evaluated by the levels of phosphorylation on Ser473 of AKT (pAKT) ± SEM. Insulin (−), basal AKT activity determined from muscle collected from 16 h fasted mice, and Insulin (+), insulin-induced AKT activity determined from muscle collected from mice 20 min post insulin injection. **A.** Images of representative western blot results. **B.** Densitometric quantification of the ratio of pAKT vs. total AKT before and after insulin injection. The value of WT mice treated with insulin and mutants with and without insulin treatment is normalized to that of WT without insulin treatment. **C.** The ratio of pAKT before and after insulin injection for each genotype. The basal pAKT was elevated but the net increase of pAKT following insulin injection was attenuated in SERT-deficient muscle. *, p<0.05, ***, p<0.001 The number of mice analyzed is indicated in parentheses.
(TIF)

Figure S5 **Quantification of plasma corticosterone levels in 3- and 6-month old mice.** Mice under normal environment were analyzed. The number of mice analyzed is indicated in parentheses.
(TIF)
