# Supplementary figures and images for: Reduced Serotonin Reuptake Transporter (SERT) Function Causes Insulin Resistance and Hepatic Steatosis Independent of Food Intake
Source: PLoS One. 2012 Mar 8;7(3):e32511. doi: 10.1371/journal.pone.0032511 (PMC3297606; doi:10.1371/journal.pone.0032511)

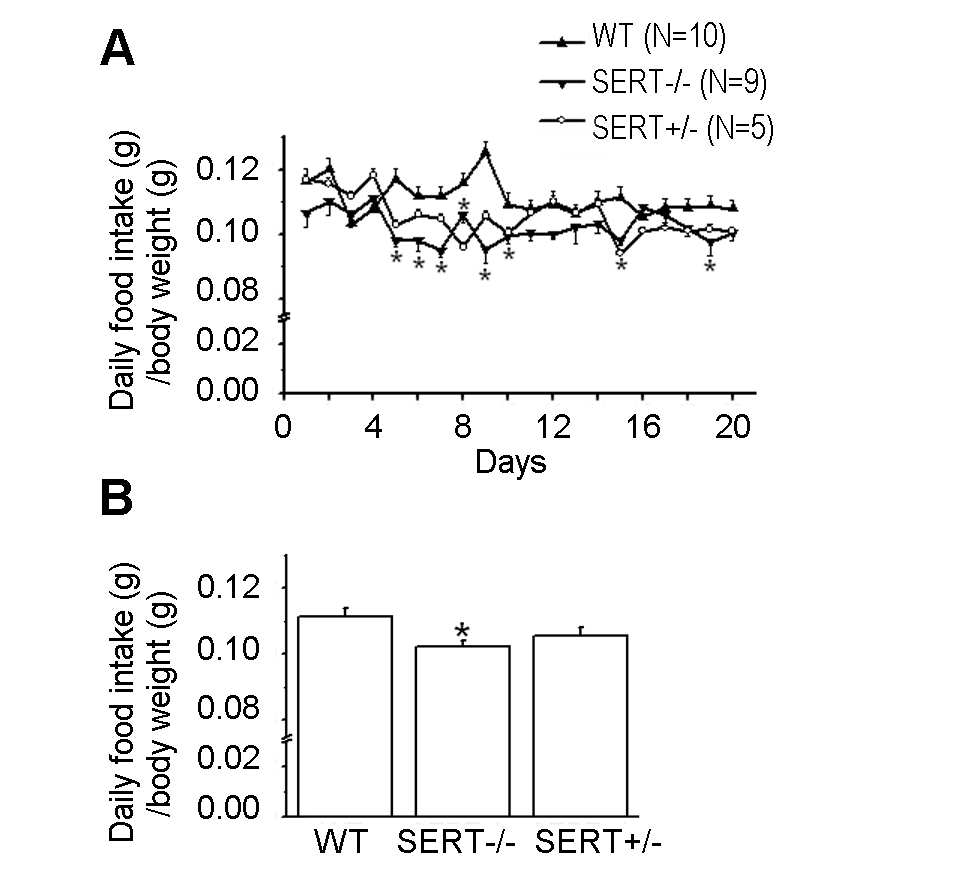

Supplement: Figure S1 — Effects of SERT deficiency on food intake. A. Daily food intake of 3-month old WT, SERT−/− and SERT+/− mice. Amount of food consumed by individually housed mice and the body weight of each mouse were monitored for 20 consecutive days. Data represent the average of daily food intake adjusted for body weight ± SEM. B. Summary of the entire monitoring period for each genotype. *, p<0.05 Student's t-test. N, number of mice analyzed. (TIF) [file pone.0032511.s001.tif]

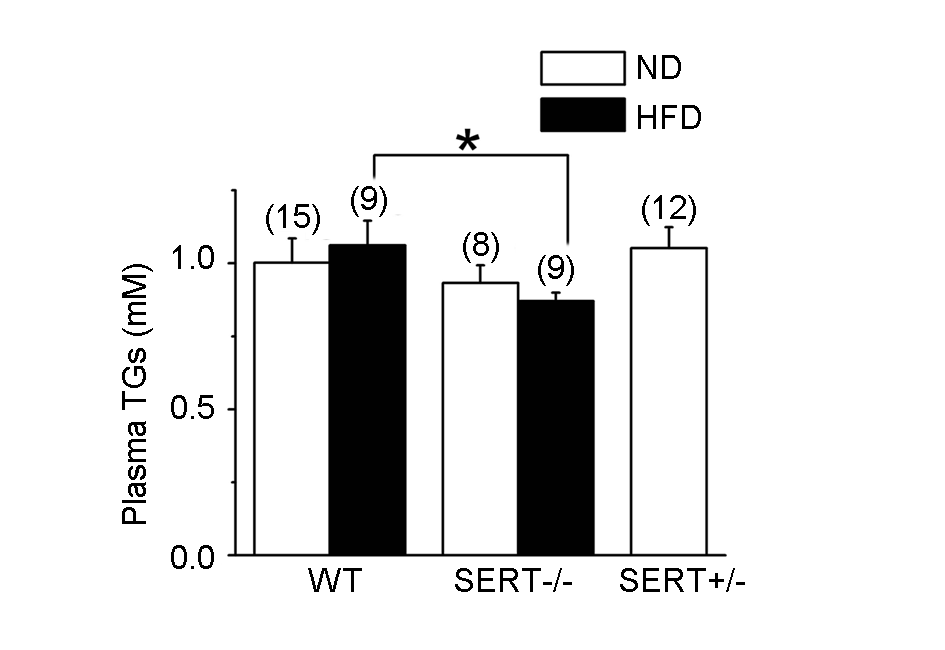

Supplement: Figure S2 — Quantification of plasma triglyceride levels in 6-month old mice fasted for 16 h. The differences between SERT mutant mice and WT mice fed ND are not statistically significant (SERT−/−, p = 0.58; SERT+/−, p = 0.66). HFD-fed SERT−/− mice exhibited lower triglyceride levels than HFD-fed WT mice, *, p<0.05 Student's t-test. The number of mice analyzed is indicated in parentheses. (TIF) [file pone.0032511.s002.tif]

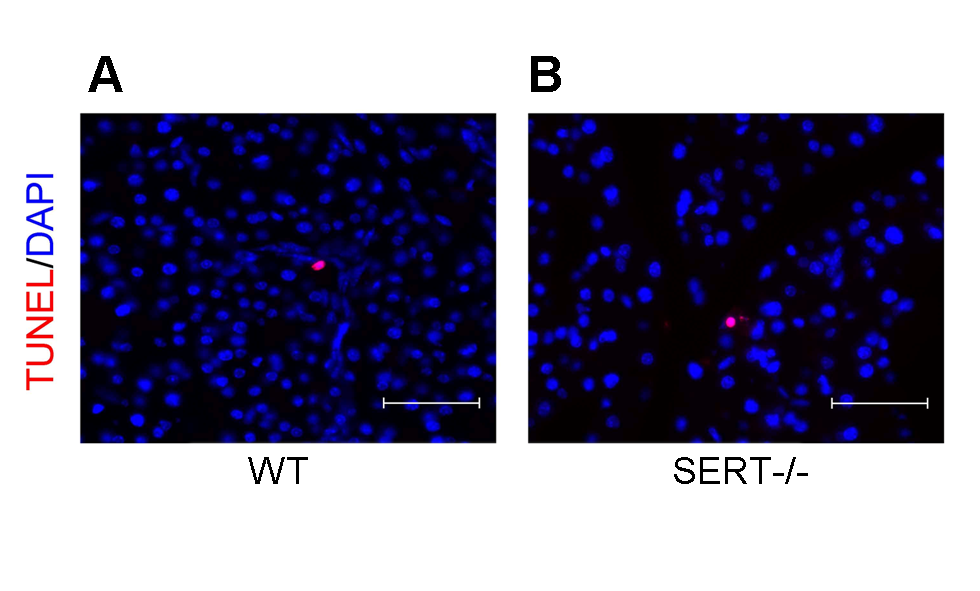

Supplement: Figure S3 — Representative photomicrographs showing TUNEL staining (red) of sections of pancreata to visualize apoptotic cells. There was no appreciable difference detected between WT and SERT−/− mice. All animals analyzed were 6-month old fed ND. Scale bar, 50 µm. (TIF) [file pone.0032511.s003.tif]

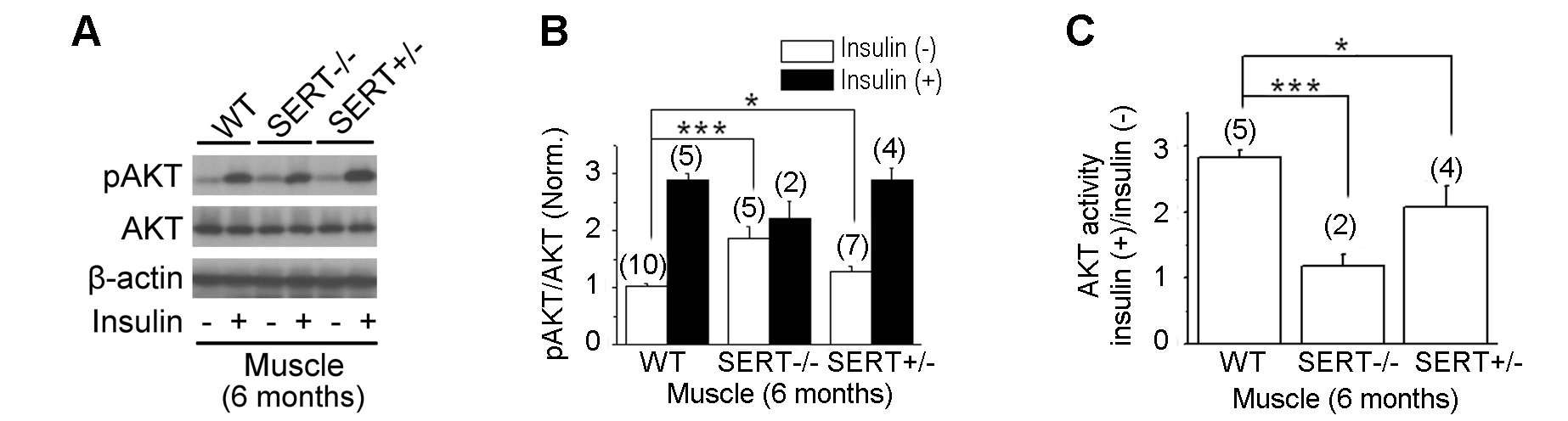

Supplement: Figure S4 — Western blot analysis of AKT activity in the muscle of 6-month old WT and SERT-deficient mice. AKT activity was evaluated by the levels of phosphorylation on Ser473 of AKT (pAKT) ± SEM. Insulin (−), basal AKT activity determined from muscle collected from 16 h fasted mice, and Insulin (+), insulin-induced AKT activity determined from muscle collected from mice 20 min post insulin injection. A. Images of representative western blot results. B. Densitometric quantification of the ratio of pAKT vs. total AKT before and after insulin injection. The value of WT mice treated with insulin and mutants with and without insulin treatment is normalized to that of WT without insulin treatment. C. The ratio of pAKT before and after insulin injection for each genotype. The basal pAKT was elevated but the net increase of pAKT following insulin injection was attenuated in SERT-deficient muscle. *, p<0.05, ***, p<0.001 The number of mice analyzed is indicated in parentheses. (TIF) [file pone.0032511.s004.tif]

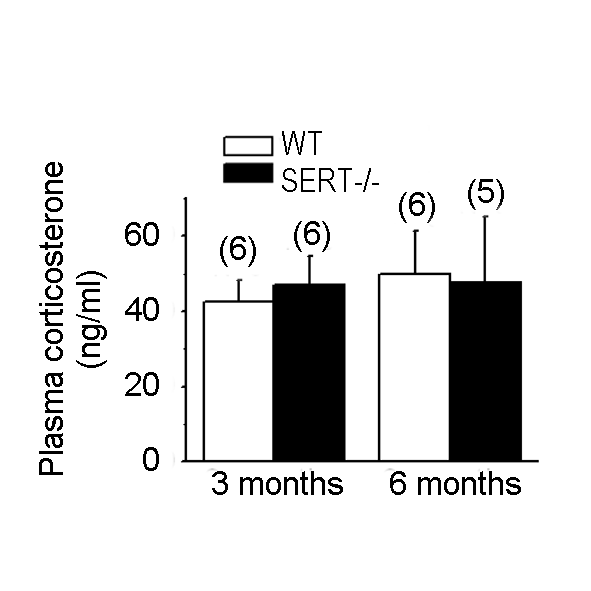

Supplement: Figure S5 — Quantification of plasma corticosterone levels in 3- and 6-month old mice. Mice under normal environment were analyzed. The number of mice analyzed is indicated in parentheses. (TIF) [file pone.0032511.s005.tif]
